# Anticancer Effect of New Cannabinoids Derived from Tetrahydrocannabinolic Acid on PANC-1 and AsPC-1 Human Pancreas Tumor Cells

**DOI:** 10.1089/pancan.2020.0003

**Published:** 2020-06-09

**Authors:** Alexander Aizikovich

**Affiliations:** AL&AM Pharmachem Ltd., Rehovot, Israel.

**Keywords:** AsPC-1, cannabinoids, *in vitro*, *in vivo*, PANC-1, THCA

## Abstract

**Purpose:** New tetrahydrocannabinolic acid (THCA) derivatives ALAM027 and ALAM108 were proposed for the treatment of the pancreatic cancer disease.

**Methods:** The *in vitro* effect of new cannabinoids ALAM027 and ALAM108 was tested against PANC-1 and AsPC-1 cell lines by CellTiter Glo assay. Pancreatic cancer xenograft model was used for the *in vivo* anticancer activity study of these compounds on PANC-1 cells.

**Results:** The *in vitro* study of new cannabinoids showed greater activity of ALAM108 than ALAM027 both for PANC-1 and AsPC-1 cells. The *in vivo* study of new cannabinoids on PANC-1 cells showed that their oral administration was effective in reducing tumor volume and tumor weight, and did not lead to any discomfort and weight loss of mice.

**Conclusion:** The cannabinoids ALAM108 and ALAM027 inhibited the tumor growing 1.6–2 times in mice with human PANC-1 cells.

## Introduction

Pancreatic cancer is one of the most dangerous forms of tumors due to its aggressive growth, early metastases, and poor response to any known therapeutic treatments. Being a fourth major cause of cancer death nowadays and with foresight to become a second cause of cancer death after lung cancer by 2030, there is an urgent need in new therapeutic strategies that might improve the disease outcome.^1^

Recently, a growing interest in the anticancer activity of cannabinoids has led to numerous studies that cover more and more types of cancer.^2,3^ Natural and synthetic cannabinoids have shown the capability to influence proliferation, migration, and apoptosis of cancer cells by both direct and indirect activation of cannabinoid receptors CB1 and CB2. In pancreatic cancer, the amount of CB1 and CB2 receptors expression in tumor cells was shown to be significantly higher than that in normal cells, opening a path for utilizing cannabinoids' anticancer capabilities to kill cancer cells without affecting normal pancreatic tissue.^4,5^

So far, the anticancer activity of cannabinoids on pancreatic tumor was addressed only in several pre-clinical studies, which showed a promising anticancer activity of tetrahydrocannabinol (THC) as well as some synthetic cannabinoids, such as WIN 55,212-2.^6^ However, looking forward to clinical stages, THC is unlikely to be used in the treatment of tumors due to its pronounced psychoactive effect. At the same time, the study of cannabinoids, having a similar structure, but lacking psychoactive properties, is of undoubted interest. One of the promising directions may be the modification of natural tetrahydrocannabinolic acid (THCA) by synthesizing its derivatives at the carboxyl group. Although THCA itself exhibits little activity against PANC-1 pancreatic cancer, the modification of a carboxylic fragment of its molecule showed a significant increase in *in vitro* inhibition of cell growth.^7^ Consequently, further modifications of the carboxyl group of THCA may lead to compounds with a more specific effect on pancreas tumor cells. The aim of this work is to study new THCA derivatives ALAM027 and ALAM108 on the pancreas PANC-1 and AsPC-1 tumor cells.

## Materials and Methods

Reagents: ALAM027 and ALAM108 were obtained from natural THCA^8^ in accordance with methods of their synthesis.^7^

*In vitro* study was performed with PANC-1 and AsPC-1 cells from the collection of Chempartner (China) by CellTiter Glo Viability Assay (cells were incubated with the compounds for 72 h at 5% CO_2_, 37°C).^5^ IC_50_ and inhibition for each compound were calculated using XLFit curve fitting software.

*In vivo* xenograft study was performed with human PANC-1 obtained from ATCC (ATCC CRL-1469™). Cells were cultured in DMEM (Cat. No. 01-055-1A; Biological Industries,), supplemented with 10% FBS (Cat. No. 10270106; Invitrogen—Life Technologies,), 1% l-glutamine (Cat. No. 03-020-1B; Biological Industries,), and 1% Pen-Strep solution (Cat. No. 03-031-1B; Biological Industries).

PANC-1 cells were thawed for 1–2 min in a 37°C bath, placed in 5 mL growth medium, and centrifuged (300 *g*, 5 min, room temperature [RT]). The cells were cultured in the growth medium. Cells were grown until 70–80% confluency before splitting. Trypsin EDTA Solution B (Cat. No. 03-052-1B; Biological Industries) was used for cell splitting, and × 2 volume of medium was used for stopping the reaction. Matrigel (basement membrane matrix, Cat. No. 3432-005-01, Lot. No. 1514944; Pathclear^®^) was thawed on ice and kept on ice until right before injection. PANC-1 cells were harvested at 70–80% confluency, centrifuged (300 *g*, 5 min, RT), and washed once more with PBS. The cells were resuspended with PBS and counted. Concentration of cells was adjusted to 2 × 10^6^/50 μL at 2 mL final volume and mixed 1:1 with 2 mL Matrigel (4 mL total). The mixture of Matrigel + cells was drawn up into a syringe (27G) just before injection. Each animal was injected with 100 μL of mixture of Matrigel + cells into the right flank.

*In vivo* testing in mice was performed in compliance with “The Israel Animal Welfare Act” and following approval of Ethics Committee of “The Israel Board for Animal Experiments”. PANC-1 cells were injected into the right flank of the mice (Athymic nude; females) at a dose of 2 × 10^6^ cells per mouse. A control group was injected with the vehicle. Tumor size was monitored using a digital caliper starting from tumor appearance (∼5 days after tumor inoculation). Doses of ALAM023 and ALAM108 were 120 and 40 mg/kg per day as solutions in canola oil accordingly based on their IC_50_ values. Solutions of cannabinoids and pure canola oil used as vehicle were given orally once a day five times a week to three groups of mice with *n* = 7 for each group. Treatment started when the tumor reached a volume of 50–80 mm^3^ in 50% of the mice (on day 32). Tumor volume was measured in female nude mice during 4 weeks of treatment with the cannabinoids. Tumor volumes in the cannabinoids-treated group were smaller already by treatment day 5, reaching statistical significance by days 19, 22, and 28 (*p < 0.05 according to two-tailed *t*-test). On study day 60, after the last tumor measurement, mice were sacrificed through CO_2_ asphyxiation. Upon termination, the tumors were excised and weighed. The average weight (Fig. 4) was also significantly lower in the cannabinoids-treated group.

## Results

**FIG. 1.**
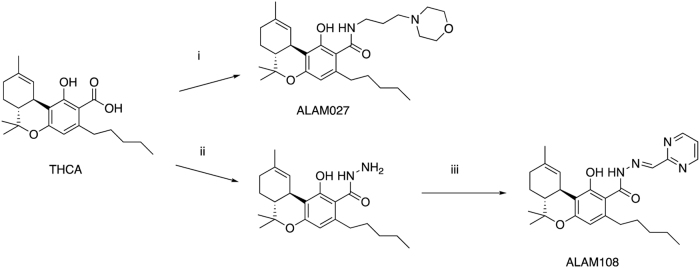
Synthesis of ALAM027 and ALAM108: **(i)** 3-morpholinopropan-1-amine, CDI, THF; **(ii)** hydrazine hydrate, CDI, THF; **(iii)** pyrimidine-2-carbaldehyde, EtOH, reflux. CDI, carbonyldiimidazole; THF; tetrahydrofuran.

The cannabinoids ALAM027 and ALAM108 containing both amide and hydrazone moieties were obtained from THCA by the following methods of synthesis (Fig. 1).^7^

**FIG. 2.**
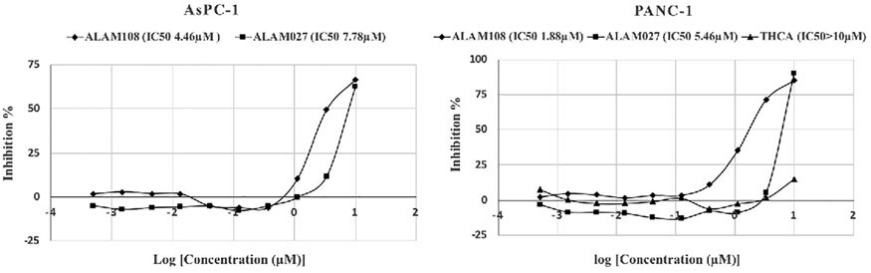
Growth inhibition curves of cells for PANC-1 and AsPC-1 at a wide range of THCA, ALAM027, and ALAM108 concentrations. THCA, tetrahydrocannabinolic acid.

*In vitro* study was designed to compare the cytotoxicity and efficacy of both cannabinoids on human PANC-1 and AsPC-1 pancreatic cancer cell lines (Fig. 2). The IC_50_ value for ALAM108 was lower than for ALAM027, THC, gemcitabine (GC) and comparable with that of nab-paclitaxel (NPT), widely used in pancreatic cancer therapy.^9,10^ Pure THCA was practically inactive (Table 1).

**Table 1.** IC_50_ and Growth Inhibition Values of Cannabinoid Compounds, THC,^6^ Gemcitabine, and Nab-Paclitaxel^a^ on PANC-1 and AsPC-1 Tumor Cells

Compounds	THCA	ALAM027	ALAM108	THC	GC	NPT
PANC-1						
Inhibition % (10 μM)	15.20	92.2	85.7	—	46	63
IC_50_ (μM)	>10	5.46	1.88	2.75	9.5	1.9
AsPC-1						
Inhibition % (10 μM)	—	64.6	67.7	—	56	32
IC_50_ (μM)	—	7.78	4.32	—	23.9	4.9

^a^ Reference.^9^

GC, gemcitabine; NPT, nab-paclitaxel; THC, tetrahydrocannabinol; THCA, tetrahydrocannabinolic acid.

**FIG. 3.**
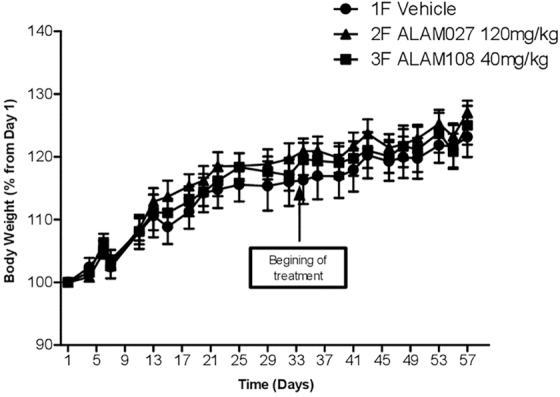
Follow-up of the mice weight throughout the study.

**FIG. 4.**
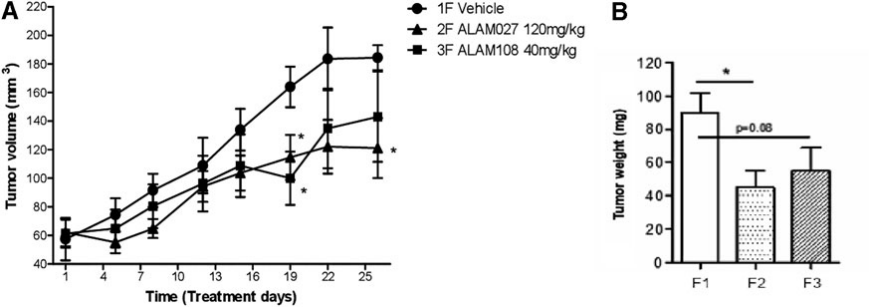
**(A)** Tumor volume measurements in female nude mice during 4 weeks of treatment with the cannabinoids 2F ALAM027 and 3F ALAM108 (*p < 0.05 according to two-tailed *t*-test); **(B)** tumor weight: F1 vehicle, F2 ALAM027 and F3 ALAM108. Results represent means ± SEM of mice in each group (statistics are presented for each group compared with Group 1F only; *p < 0.05 according to *t*-test). SEM, standard error of the mean.

*In vivo* study was performed by the pancreatic cancer xenograft model in nude female mice. The weight of mice was monitored three times a week (Fig. 3). As can be seen, all mice gained weight steadily during the study, with no significant difference between cancer cells treated mice versus vehicle injected mice. The average weight of the mice upon study initiation was 19.7 g and that upon treatment initiation was 23.6 g. The tumor size was monitored using a digital caliper twice a week from day 32 after inoculation (day 1 of treatment) till day 28 of treatment, and the tumor volume was calculated using the equation *V* = *LW*^2^/2, where *L* is the length and *W* the width (Fig. 4A).

Tumor volume measurements in female nude mice were carried out during 4 weeks of treatment with the cannabinoids. The volumes in the treated groups were smaller already by treatment day 5, reaching statistical significance by days 19, 22, and 28. On study day 60, after the last tumor measurement, mice were sacrificed through CO_2_ asphyxiation. Upon termination, the tumors were excised and weighed. As shown in Figure 4B, the average tumor weight in cannabinoids-treated groups was 1.6–2 times lower than that in the vehicle group.

## Discussion

It is known that PANC-1 and AsPC-1 are types of pancreatic tumors that are difficult to treat with known anticancer drugs. A comparison of the *in vitro* activity of THC as well GC and NPT,^6,9,10^ widely used for this purpose, with the results obtained in this study shows that they are quite close (Table 1). *In vivo* data obtained for ALAM027 and ALAM108 for PANC-1 (Figs. 3 and 4) can also be close to these data for THC, GC, and NPT both by the ability to inhibit tumor growth and by the effect on the physical condition of animals during the study. At the same time, these cannabinoids differ favorably in the manner of drug administration (orally for cannabinoids and parenterally for THC, GC, and NPT), which makes their use more convenient in the treatment of tumors. Of interest is also the relationship between the doses of cannabinoids, the degree of *in vivo* inhibition of tumor growth, and IC_50_ values, which may indicate both same bioavailability of these compounds and a similar mechanism of action.

## Conclusion

The *in vitro* study of new cannabinoids showed greater activity of ALAM108 than of ALAM027 both for PANC-1 and AsPC-1 pancreas tumor cells. The *in vivo* study of these cannabinoids on PANC-1 cells showed that their oral administration decreased the tumor size 1.6–2 times and did not lead to any discomfort, psychotic effects, and weight loss of mice. Further study of these compounds will allow to determine the mechanism of their action on cancer cells and may open the way to new therapeutic drugs based on THCA.

## Abbreviations Used

GC: gemcitabine
NPT: nab-paclitaxel
RT: room temperature
THC: tetrahydrocannabinol
THCA: tetrahydrocannabinolic acid
